# Real Time Generation of Three Dimensional Patterns for Multiphoton Stimulation

**DOI:** 10.3389/fncel.2021.609505

**Published:** 2021-02-24

**Authors:** Paolo Pozzi, Jonathan Mapelli

**Affiliations:** ^1^Department of Beiomedical, Metabolic and Neural Sciences, University of Modena and Reggio Emilia, Modena, Italy; ^2^Center for Neuroscience and Neurotechnology, University of Modena and Reggio Emilia, Modena, Italy

**Keywords:** multiphoton microcopy, wavefront control, optogenetics, computer generated holograms, spatial light modulators, GPU (CUDA)

## Abstract

The advent of optogenetics has revolutionized experimental research in the field of Neuroscience and the possibility to selectively stimulate neurons in 3D volumes has opened new routes in the understanding of brain dynamics and functions. The combination of multiphoton excitation and optogenetic methods allows to identify and excite specific neuronal targets by means of the generation of cloud of excitation points. The most widely employed approach to produce the points cloud is through a spatial light modulation (SLM) which works with a refresh rate of tens of *Hz*. However, the computational time requested to calculate 3D patterns ranges between a few seconds and a few minutes, strongly limiting the overall performance of the system. The maximum speed of SLM can in fact be employed either with high quality patterns embedded into pre-calculated sequences or with low quality patterns for real time update. Here, we propose the implementation of a recently developed compressed sensing Gerchberg-Saxton algorithm on a consumer graphical processor unit allowing the generation of high quality patterns at video rate. This, would in turn dramatically reduce dead times in the experimental sessions, and could enable applications previously impossible, such as the control of neuronal network activity driven by the feedback from single neurons functional signals detected through calcium or voltage imaging or the real time compensation of motion artifacts.

## 1. Introduction

The recent advances in the field of photonics (Pozzi et al., 2015) combined with methods of molecular (Gandolfi et al., 2017) and genetic manipulation of the samples (Boyden et al., 2005; Mutoh et al., 2012), have provided novel tools to investigate neural functions. Among these tools, optogenetics allows to selectively stimulate specific neuronal subtypes within a three-dimensional sample (Packer et al., 2013). Indeed, in order to avoid the stimulation of undesired out-of-focus cells, multiphoton stimulation is required (Papagiakoumou et al., 2010; Dal Maschio et al., 2017). The near-simultaneous stimulation of multiple cells heterogeneously distributed in three dimensions can be achieved by time multiplexing with high-speed, inertia-free scanners (Wang et al., 2011), but the only known method for truly simultaneous stimulation is the use of spatial light modulators (SLM) (Packer et al., 2012).

A coherent light source can be focused simultaneously in an arbitrary pattern of diffraction limited focal points within a three-dimensional volume through the use of a spatial light modulator in the pupil of an optical system. In order to stimulate areas wider than the diffraction limit, the technique can be combined with either temporal focusing (Pégard et al., 2017), or spiral or raster scanning (Packer et al., 2012, 2013). While this method is widely used in optogenetics, it has a variety of applications extending beyond the field of neuroscience and including optical trapping (Grier and Roichman, 2006), high throughput spectroscopy (Nikolenko et al., 2008; Gandolfi et al., 2014; Pozzi et al., 2015), and adaptive optics (Pozzi et al., 2020).

A recent publication (Zhang et al., 2018) showed how multiphoton optogenetics, applied in conjunction with multiphoton calcium imaging, can be used to manipulate in real time a network of neurons, for example clamping their calcium activity to a given threshold, or forcing cells to co-activate. However, due to the limitations in pattern calculation speeds, the method can only control the stimulation by alternating amongst a limited amount of pre-calculated patterns. True, real-time feedback-based control of a network would be greatly enhanced by the ability of calculating patterns automatically on-the-fly as they are needed.

The requirements for real-time optogenetics manipulation of calcium signals can vary widely depending on the optical setup, experiment goals, species of interest, cell type, and brain region. For the number of cells of interest and their distribution, at the state of the art for *in vivo* imaging, random access multiphoton microscopy was shown to be able to acquire signals from over five hundred cells, within an approximately 300μm fov in all three directions at 80 Hz (Katona et al., 2012) in visual cortex. Other implementations showed performance in the same orders of magnitude, for example Bessel scanning (Lu et al., 2017) showed the activity of approximately one hundred GABA-ergic neurons at 30 Hz in the same region. In alternative samples and technologies, lightsheet microscopy in Zebrafish embryos (Wolf et al., 2015) was shown to detect signals from tens of thousands of neurons at 1 Hz from the whole embryo brain, and its acquisition frequency could increase dramatically by reducing the field of view.

As for the time resolution requirements, it mainly depends on the accuracy required for the cell response to photostimulation, as well as from the rise and fall time of calcium signals in the neurons of interest. Those in turn strongly depend on the dye or protein used for calcium imaging and on the cellular type of the neurons stimulated. Rise times are known to be generally really fast when photostimulation is activated, reaching a saturation of the signal within a couple of hundreds milliseconds. As for decay times, they are generally in the order of a second, but can go down to a few hundreds milliseconds in some transgenic mice lines (Dana et al., 2014). Even in the assumption of a calcium signal decreasing quite slowly with an exponential decay time of 1 s (corresponding to a complete return to baseline fluorescence in approximately three seconds), a signal decrease of 10% happens in the first 100 ms, which indicates the need for SLM modulation frequencies higher than 10 Hz for good optogenetic clamping of the activity. At the very limit of such scenario, cerebellar granule cells bulk stained with Fura-2 AM dye have been shown to have, under electrical stimulation, calcium transients shorter than 200 ms from the onset to the return to baseline (Gandolfi et al., 2014), and would therefore require millisecond-scale modulation of the stimulation pattern for real-time control.

While the fields of view typical of high speed 3D calcium imaging are generally within the operating capabilities of modern SLMs, targeting hundreds of neurons with milisecond-scale modulation is a challenging endeavor. While high performance SLMs can refresh at up to hundreds of *Hz*, the algorithms used for computing holograms constitute the current main limitation.

For two dimensional patterns, or patterns distributed on a limited set of two-dimensional planes, relatively fast computation times can be achieved by exploiting fast Fourier transform based algorithms (Sinclair et al., 2004). However, the generation of an arbitrary 3D pattern remains the main limiting factor in the speed of operation for spatial light modulators, slowing the entire experimental procedure, and precluding any form of real-time update of three dimensional patterns. The generation of a three dimensional focusing pattern requires estimation of the phase value for each of the hundreds of thousands of pixels of the spatial light modulator maximizing the quality of the obtained pattern. The two most popular algorithms for this computation are the high-speed, lower precision random superposition (RS) algorithm, and the higher precision, lower speed Weighted Gerchberg-Saxton (WGS) algorithm (Di Leonardo et al., 2007). The RS computational cost scales linearly with *M* · *N*, where *M* is the number of SLM pixels and *N* is the number of generated foci, while WGS scales linearly with *M* · *N* · *I*, where *I* is the number of iterations required. The quality of the hologram is generally evaluated through its efficiency (*e*) and uniformity (*u*), two metrics respectively indicating as a number between 0 and 1, the percentage of laser light actually focused in the desired locations, and the uniformity of intensities between the generated foci.

At the state of the art, when implemented with a typical SLM resolution on a consumer computer processor unit (CPU), RS can generate holograms with *e* > 0.2 and *u* > 0.2 in a few seconds, while WGS can generate holograms with *e* > 0.9 and *u* > 0.9. Unfortunately, WGS requires a few minutes for computation. Since most applications require faster computation times, it is crucial to implement such algorithms on faster time scales as it has been obtained by using a consumer graphical processors (GPU) (Bianchi and Di Leonardo, 2010). When implemented on a GPU, RS algorithm has been proved to promptly generate arbitrary patterns at video rate (Reicherter et al., 2006; Daria et al., 2009), but with its characteristic low quality. Conversely, the WGS algorithm has proven to produce high quality holograms at video rate, but only with a limited number of SLM pixels (*M* < 768^2^) and on a very low number of foci (*N* < 10) (Bianchi and Di Leonardo, 2010; Vizsnyiczai et al., 2014). Additionally, although WGS results were published, no source code was openly released with them. As a result, due to the intrinsic difficulty in GPU coding, this profitable method has not yet been widely adopted, and most researchers still perform WGS computation on CPUs.

We have recently proved (Pozzi et al., 2019), how, on a CPU, a new algorithm (compressive sensing weighted Gerchberg-Saxton, CS-WGS), applying the principles of compressed sensing to the iterations of WGS can reduce its computational cost asymptotically close to the cost of RS, while maintaining the high quality of WGS holograms. Here, we present the implementation of CS-WGS on a low-cost consumer GPU, demonstrating that the algorithm is well-suited to GPU implementation, enabling video-rate computation of holograms with *e* > 0.9 and *u* > 0.9 for *N* < 100 and *M* < 1, 152^2^, ideally adaptable to feedback-based optogenetic control of neuronal networks.

## 2. Methods

### 2.1. Compressive Sensing Weighted Gerchberg Saxton Algorithm

In both RS and WGS algorithms, the SLM phase pattern Φ^0^(*x*′, *y*′) generating a set of *N* foci at positions *X*_*n*_ = {*x*_*n*_, *y*_*n*_, *z*_*n*_} with relative intensities ${\left\|a_{n}^{0}\right\|}^{2}$, is calculated as the phase of the interference of the *N* wavefronts with known phase patterns $\phi_{n}\left(x^{\prime },y^{\prime }\right)$ generating each spot independently, each with a set phase delay $\theta_{n}^{0}$:

(1) $$\begin{array}{l} \Phi^{0}=\text{arg}\left(\overset{N}{\underset{n=1}{\sum }}a_{n}^{0}e^{i\left(\phi_{n}+\theta_{n}^{0}\right)}\right) \end{array}$$

where ϕ_*n*_ is defined by basic physical optics as:

(2) $$\begin{array}{l} \phi_{n}\left(x^{\prime },y^{\prime }\right)=\frac{2\pi }{\lambda f}\left(x_{n}x^{\prime }+y_{n}y^{\prime }\right)+\frac{2\pi }{\lambda f^{2}}\left({x^{\prime }}^{2}+{y^{\prime }}^{2}\right)z_{n} \end{array}$$

In the simple random superposition algorithm, Φ^0^ is simply determined through Equation (1), selecting random values for $\theta_{n}^{0}$. In the weighted Gerchberg-Saxton algorithm, the values of θ_*n*_ are determined through a series of alternating projections between the SLM space and the spots' positions. The algorithm begins by computation of the RS hologram Φ^0^ through Equation (1). At the *j*-th iteration, the field $E_{n}^{j}$ of each spot is calculated as:

(3) $$\begin{array}{l} E_{n}^{j}=\underset{x^{\prime },y^{\prime }\in \Omega }{\sum }Ae^{-i\left(\Phi^{j-1}-\phi_{n}\right)} \end{array}$$

where ‖*A*(*x*′, *y*′)‖^2^ is the distribution of light intensity at the slm surface, and Ω is the set of all SLM pixels coordinates. At this point the values of θ_*n*_ and *a*_*n*_ are updated as:

(4) $$\begin{array}{l} w_{n}^{j}=w_{n}^{j-1}\frac{{\langle \left\|E_{n}^{j-1}\right\|\rangle }_{n=1}^{N}}{\left\|E_{n}^{j-1}\right\|} \end{array}$$

(5) $$\begin{array}{l} a_{n}^{j}=w_{n}^{j}a_{0} \end{array}$$

(6) $$\begin{array}{l} \theta_{n}^{j}=\text{arg}\left(E_{n}^{j-1}\right) \end{array}$$

where $w_{n}^{j}$ are weight factors, all initialized at 1 for the first iteration. The updated values of $a_{n}^{j}$ and $\theta_{n}^{j}$ are used to compute a new hologram Φ^*j*^ with Equation (1) and start the next iteration.

The CS-WGS algorithm is equivalent to WGS, but the summation in Equation (3) is only performed over a subset $\Omega_{\text{compressed}}^{j}$ of randomly distributed pixels on the SLM for *N* − 2 iterations, followed by two full iterations to ensure full convergence and the computation of phase on all SLM pixels. Conversely the value of the hologram phase can be computed, for all iterations except the last two, only for the pixels in $\Omega_{\text{compressed}}^{j}$. Through this adaptation, CS-WGS scales in computational cost linearly with 2 · *M* · *N* + *c*(*M* · *N* · (*I* − 2)), where *c* is the ratio between the sizes of $\Omega_{\text{compressed}}^{j}$ and Ω.

The performance of all three described algorithms can be computed through the metrics of efficiency (*e*), uniformity (*u*), and variance (*v*). Efficiency is computed as the fraction of power effectively directed at the spots locations:

(7) $$\begin{array}{l} e=\underset{n}{\sum }I_{n} \end{array}$$

where *I*_*n*_ is the fraction of laser intensity directed to the *n*-th spot. The uniformity metric is defined as:

(8) $$\begin{array}{l} u=1-\frac{{max}_{n}\left(F_{n}\right)-{min}_{n}\left(F_{n}\right)}{{max}_{n}\left(F_{n}\right)+{min}_{n}\left(F_{n}\right)} \end{array}$$

where *F*_*n*_ is the ratio between the achieved and desired power fractions at the *n*-th spot:

(9) $$F_{n}=\frac{I_{n}}{\sum_{n^{\prime }}I_{n^{\prime }}}/\frac{\|a_{n}^{0}{\|}^{2}}{\sum_{n^{\prime }}\|a_{n^{\prime }}^{0}{\|}^{2}}$$

Finally, the variance metric is expressed as the mean square relative error in the power fractions:

(10) $$v=\frac{\sum_{n}{\left(F_{n}-1\right)}^{2}}{N}$$

The efficiency metric reports on the actual fraction of power directed to the spots. It should be noted that the power fraction not directed to the spots is rarely uniformly distributed throughout the sample, and generally forms undesired excitation spots. The metric should therefore be as close to the value of 1 as possible to avoid undesired artifacts, and low values can not only be compensated by an increase in laser power.

The uniformity metric should also be as close to 1 as possible. Lower values reveal the presence of significant outliers in the spots intensities, which can lead to missing excitation of targeted cells, or to local photodamage in over-illuminated cells. Finally, the variance metric defines the general deviation of spots intensities from their desired values, and should be as close to 0 as possible in order to achieve precise control of power over all generated spots. Precise control of intensities is crucial for optogenetics stimulation, as the relative power between spots should be carefully regulated in order to prevent non-optically sectioned stimulation due to thermal effects (Picot et al., 2018).

### 2.2. GPU Implementation

GPU implementations of algorithms should be carefully developed in order to fully exploit the parallelized calculation performance of the devices. We report here some considerations about the implementation.

#### 2.2.1. Global Memory Allocation

When implementing GPU code, minimization of memory transfer between the system memory and the GPU global memory is critical to achieve optimal performances. RS, WGS, and CS-WGS are all very well suited algorithms for this specific requirement, as the hologram specific inputs required are limited to the 3D coordinates of the desired spots and their desired intensities, as well as a single floating point value for the required compression factor *c* for CS-WGS. As most SLMs are connected to calculators as secondary monitors directly connected to the GPU, no readout of the algorithm's output to system memory is necessary, but the hologram is directly projected on the SLM through CUDA-OPENGL interoperability.

Additionally, some fixed parameters characterizing the physical and geometrical properties of the SLM and the optical system (e.g., the coordinates *x*′, *y*′ of the SLM pixels, the phase to gray scale lookup table of the SLM output), are uploaded to the GPU only once at startup and used for all holograms computed during an experimental session. Such initialization does not therefore affect the speed of the algorithm convergence.

#### 2.2.2. Backwards Propagation of RS and WGS

Given, for each spot, the values of the desired coordinates and intensities *X*_*n*_, $a_{n}^{0}$, weights $w_{n}^{j}$ and phase terms $\theta_{n}^{j}$, at each iteration the hologram phase is computed according to Equation (1). Each of the parallel threads of the GPU evaluates the equation for one of the *M* pixels of the SLM, performing the summation over all spots. Counter-intuitively, the values of ϕ_*n*_ are computed at each iteration according to Equation (2), instead of computed once and stored in global memory, as their direct computation is significantly faster than accessing values stored in the GPU global memory.

The obtained hologram Φ^*j*^ is stored in a pre-allocated section of global memory, or, in case of the last iteration, copied to an OpenGL texture buffer, and projected on the SLM surface. It should be noticed that vertical synchronization in the OpenGL environment should be enabled, in order to avoid artifacts during the alternation of different holograms on the SLM. As a consequence, the total time required for the last iteration will be extended until the next refresh of the SLM screen.

#### 2.2.3. Forward Propagation of RS and WGS

Given an hologram Φ^*j*^, and the known intensity distribution of light at the SLM surface, the field at each spot can be computed through Equation (3), which therefore requires the sum of *M* complex numbers per each spot. This sort of computation is known in GPU programming as a dimensionality reduction, and is performed by using *k* threads to iteratively perform the sum of *M*/*k* elements of the sum, until the amount of elements to be summed equals one. Since a modern GPU can run 1,024 threads in one block, and the number of SLM pixels in the system aperture is <1,024^2^, the dimensionality reduction always converged in two iterations for the presented results.

#### 2.2.4. Compressed Sensing

During initialization, all arrays containing data referring to SLM pixels (e.g., hologram phase, known intensity at the pupil) are reorganized in a randomly selected order. At each iteration only *c* · *M* GPU threads are employed both for forwards and backwards projection, performing computation on pixels which will be adjacent in GPU global memory for optimal performance, but randomly distributed in the pupil due to the random reorganization. Only the backwards projection at the very last iteration is performed on all pixels, in order to compute the phase of the full hologram. The actual position in the pupil for each pixel is stored during initialization in an additional array in global memory, and used at the end of the computation to apply the correct phase values to the correct OpenGL texture pixels for projection.

### 2.3. Experimental Setup

Holograms were computed on a budget desktop GPU (GTX1050, Nvidia), also available in several mid-range laptops. Experimental results were obtained by measuring two-photon excited fluorescence from a solid, 1.7 mm thick fluorescent slide (FSK-2, Thorlabs, USA) on a custom system for multiphoton imaging and optogenetics. The system includes an SLM with a refresh frequency of 31 Hz, and a panel of 1,152 × 1,920 pixels, with pixel pitch of 9.2μm (Meadowlark, USA), with the short side optically matched to the round aperture of the optical system, limiting hologram computation to a round sub-region of 1,152 pixels in diameter.

**Figure 1:**
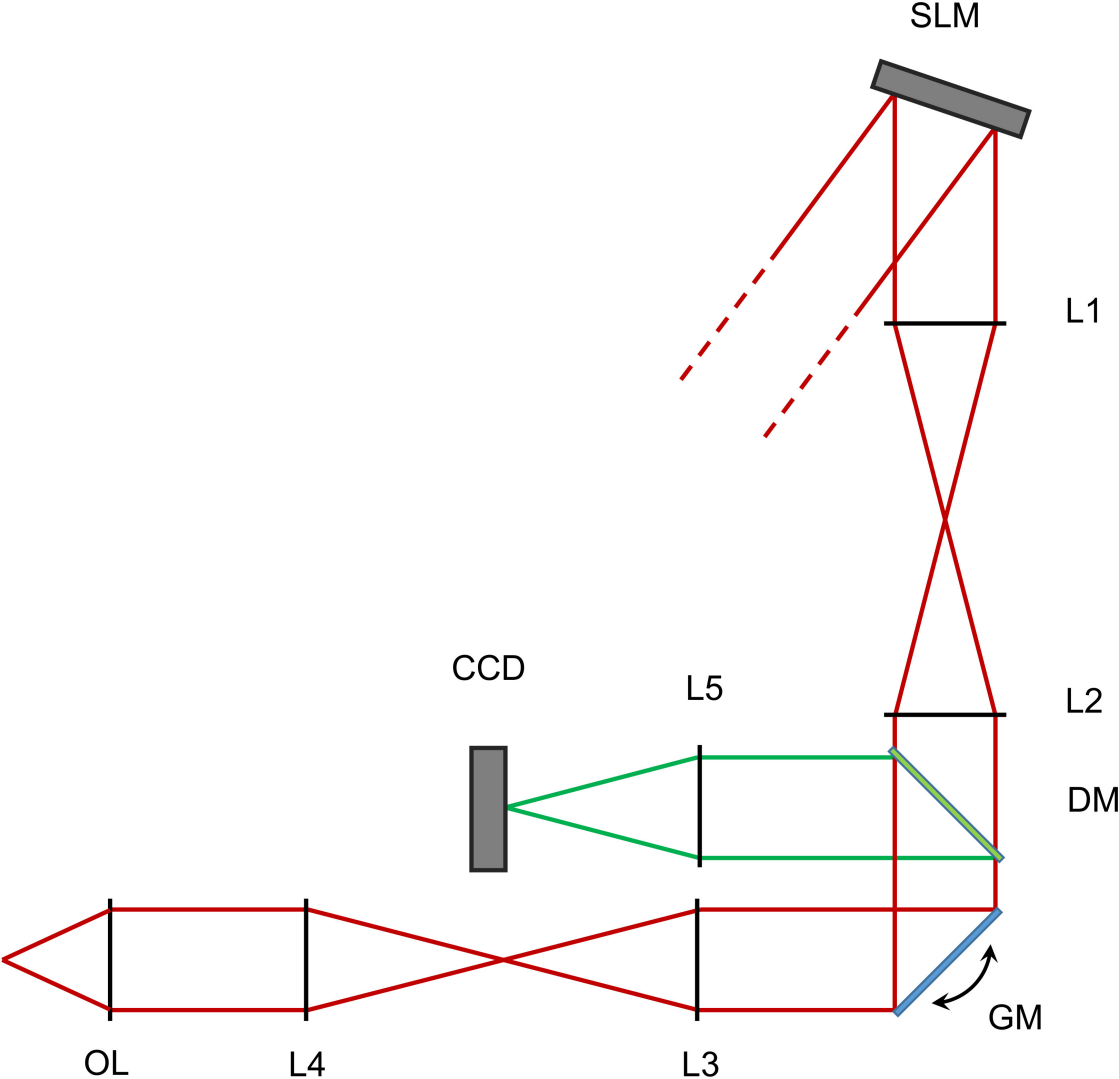
Scheme of the optical setup for the reported experiments. Not to scale. Red lines show the excitation light path, green lines represent the fluorescent light path after descanning. SLM - Spatial light modulator. L1-L2 - First 4f telescope. GM - Galvanometric mirrors. L3-L4 - Second 4f telescope. OL - Objective lens. DM - Dichroic mirror. L5 - Focusing lens. CCD - Detector camera.

The source employed is a Ti:Sa laser (Chameleon Ultra II, Coherent, USA), tuned to 800 nm, expanded through a telescope of two infrared achromatic doublets (AC-127-050-B and AC-254-250-B, Thorlabs) to a beam waist radius of 6 mm at the SLM panel. A simplified scheme of the setup is shown in Figure 1.

The spatial light modulator (SLM) surface is conjugated to a couple of silver coated galvanometric mirrors (GM, GVS-012/M, Thorlabs, USA) by a 4-f beam reducing telescope of two infrared achromatic doublets (L1 and L2, AC-508-200-B and AC-508-150-B, Thorlabs). A custom made glass slide with a 0.5 mm round deposition of titanium is placed in the focal plane of the first lens in order to block the 0-th order of diffraction of the SLM while minimally affecting the projected pattern. We were in fact unable to measure any differences in spots intensities when adding and removing the blocker. The Galvanometric mirrors are conjugated through a beam expanding 4-f telescope of broad spectrum achromatic doublets (L3 and L4, AC-508-180-AB and AC-508-400-AB) to the back aperture of a water dipping microscope objective (OL, XLUMPlanFL N, 20X, 1.0 NA, Olympus, Japan). In this configuration, a phase-conjugated image of the SLM is produced on the back aperture of the objective with a magnification of 5:3, so that the 10.6 mm side of the SLM is matched with the 18 mm aperture of the objective.

Fluorescence light is reflected by a longpass dichroic mirror (DM, FF665-Di02-25x36, Semrock, USA) and further filtered from laser light through an IR-blocking filter (FF01-680/SP-25, Semrock, USA). The mirrors are conjugated by a couple of 4-f telescopes of visible achromatic doublets and a custom channel splitter (not shown) with a mounted 12 − 72 mm, 1.2*f#* zoom lens (L9, Cosina, Sony, Japan), mounted on a high speed, 128 × 128 pixels EMCCD camera (CCD, Hnu 128 AO, Nuvu, Canada).

The focal and aperture of the camera zoom lens are chosen in order to image a field of view of 400μm × 400μm for two color channels in 64 × 64 pixels subregions of the camera sensor, while maintaining a depth of field of 400μm in order to visualize three-dimensional patterns without defocus aberrations. Focusing of the laser in the fluorescent slide generates two-photon fluorescence, the intensity of which increases quadratically with local power, and is therefore an appropriate reporter of the stimulation intensity which could be achieved in a biological sample.

Measurements were performed at approximately 300μm depth within the fluorescent slide, in order to avoid spots generated at high axial distances from the focal plane to be focused outside the sample. The galvanometric mirrors were operated in a 50μ*m* wide constant speed spiral scan at 120 Hz throughout the experiments, in order to minimize photobleaching effects, as well as compensating for local inhomogeneities of the fluorescent slide. The descanned nature of the detection light path insured that the motion of the mirrors did not affect the shape of the spots at the detector.

## 3. Results

**Figure 2:**
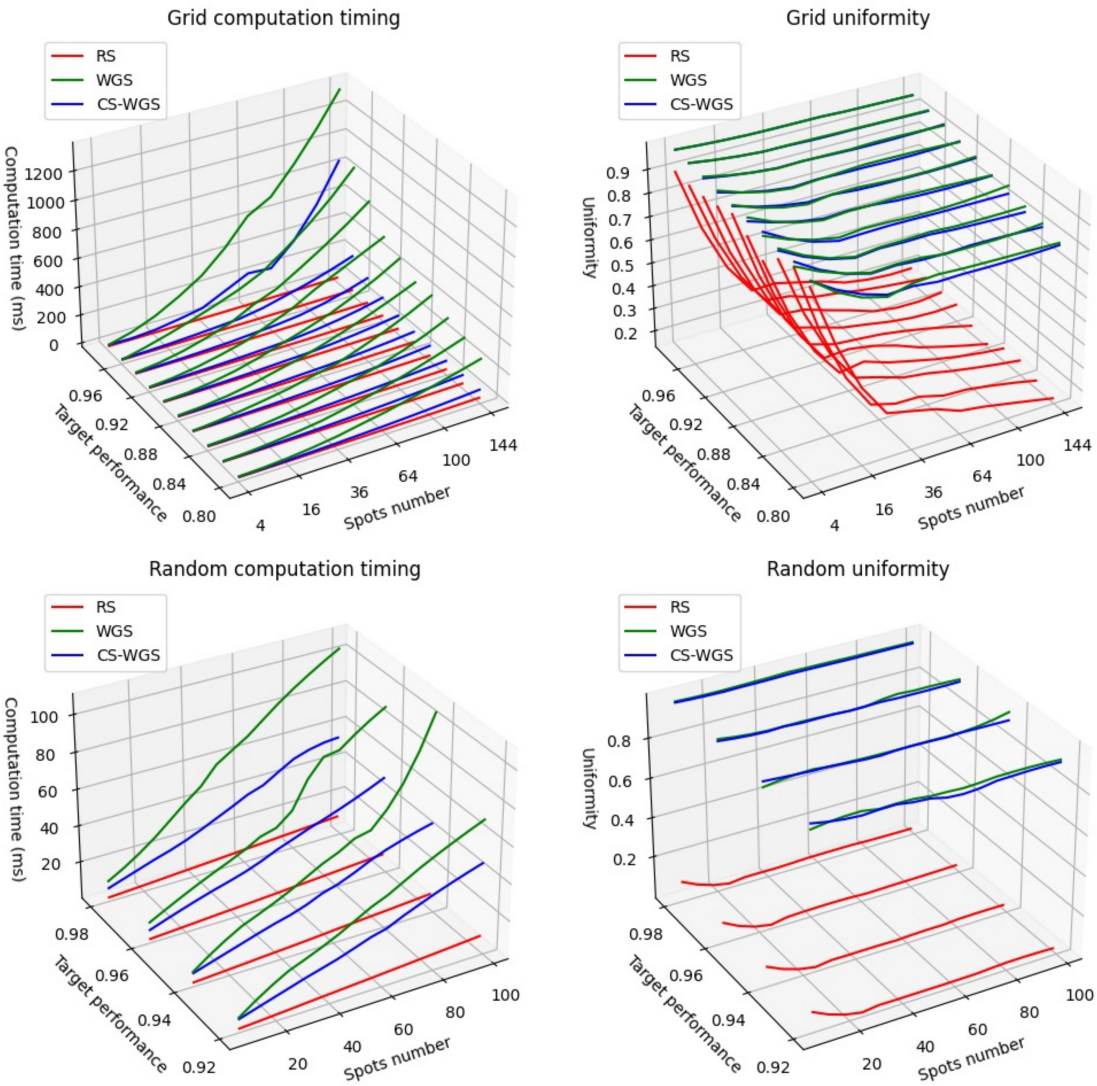
Performance comparison of the algorithms when computing patterns at a set fraction of the full convergence uniformity.

In order to compute convergence timing for RS, WGS, and CS-WGS algorithms, two types of holograms were computed: regular two-dimensional grids of uniform spots, considered as a worst case scenario for pattern uniformity, and a more realistic random distributions of spots of varying intensity within a cubic volume of 200μm. Grids were calculated for square patterns from 4 to 144 spots. Random distributions were calculated from 9 to 99 spots. Lower amounts of spots were not considered, as SLMs have generally unreliable performance independently from the algorithm used when generating very few spots. If possible, in such situation, other excitation methods should be preferred (e.g., acousto-optic scanners). A maximum performance reference was computed through 200 iterations of WGS. Holograms for the same distributions of points were then calculated with RS, with WGS, and with CS-WGS for compression factors ranging from 2^−1^ to 2^−8^. WGS and CS-WGS computations were repeated for an increasing number of iterations, until a uniformity value higher than a target percentage of the maximum performance was reached. Figure 2 shows the timings required for full convergence of the algorithms, as well as a comparison between the uniformity performances achieved by the non-iterative RS compared to the iterative algorithms. Only the best performing value of the compression factor in CS-WGS is reported for each data point. For these results, vertical synchronization of the GPU with the SLM screen was disabled, in order to present data unaffected by the specific hardware employed. The data reported clearly shows how, in any of the presented scenarios, CS-WGS greatly outperforms WGS, with generally half the convergence time, and up to a factor 5 speedup when computing holograms for regular lattices of high numbers of spots. This, while being an unlikely pattern for optogenetics experiments, is often required for imaging or optical trapping applications.

While still significant, the lowest performance advantage of CS-WGS over WGS, was observed for random distributions of small numbers of spots (<50) for relatively low performance targets (<92% of full convergence uniformity) for which WGS converged in only two iterations, leaving small space for improvement with the application of compressed sensing. In this situation, WGS still resulted 1.5 times slower than CS-WGS.

It should be noticed how, while a GPU implementation of RS remains up to an order of magnitude faster than iterative algorithms, the uniformity of the patterns produced can be extremely low for any number of spots, and this algorithm should only be used when the experimental scenario requires extremely high computation speed for a very high number of spots.

A more realistic utilization scenario for high speed hologram computation, however, is one in which the full convergence performance is sacrificed in order to achieve computation times equivalent to the refresh rate of the SLM, in order to update the hologram on-the-fly as fast as the hardware allows it. Fixed refresh rate performance of RS, WGS, and CS-WGS algorithms was measured both through calculation of the theoretical efficiency and uniformity of the patterns, and by visualization of multiphoton fluorescence excitation in the experimental setup. In these measurements, vertical synchronization of the GPU with the SLM screen was enabled, as it is required for correct experimental application. The SLM used for data validation was capable of a refresh rate of 31 Hz. However, hologram computing times were constrained to a refresh rate of 15 Hz, as it was experimentally found that, while operating at the SLM limit of 31 Hz, the quality of the projected pattern was strongly dependent on the pixel response times of the SLM at the experimental wavelength, and comparison of experimental data resulted difficult. The performance of CS-WGS was computationally tested for a range of compression rates *c* from 2^−1^ to 2^−8^. The best performing compression rate for the uniformity metric was used for experimental comparison. An additional set of measurements for full convergence of WGS was added in order to provide a reference for the best achievable pattern quality without frame rate constraints.

Tests were performed in three critical scenarios for multi-foci real-time computation. The first two were two-dimensional, regularly spaced, grids of points rotating in 3D space, representing a worst-case scenario for pattern uniformity. The two grids differ in number of total spots, one is a grid of 100 spots, for which WGS could only perform a single iteration within the 64 ms frame time limit, the other is a more limited 36 spots grid, for which WGS could achieve 5 full iterations. The third scenario was a more realistic distribution of 100 points in a random pattern, within a cubic volume of side 300μm, with randomly distributed target intensities.

**Figure 3:**
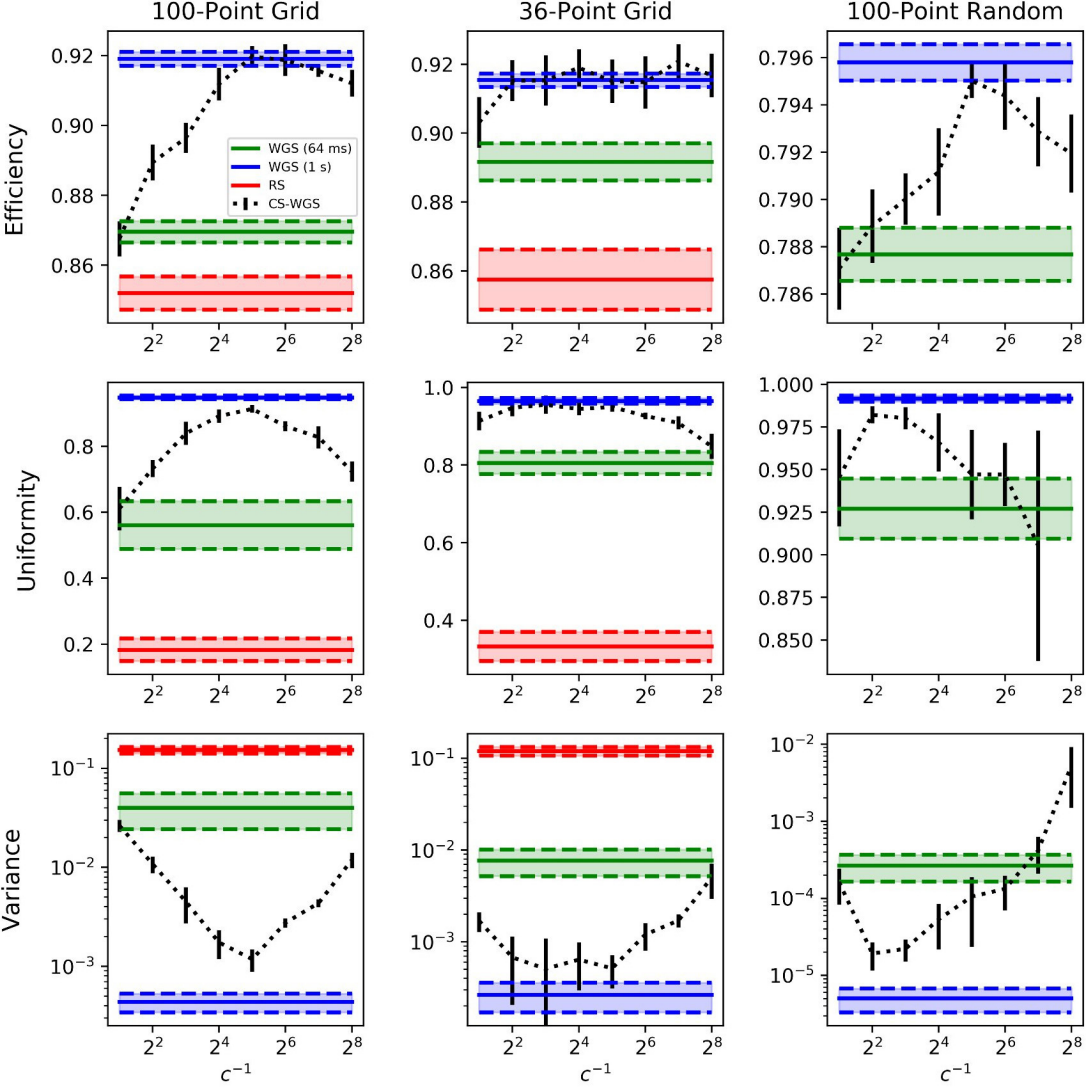
Performance comparison of the algorithms when computing in real time at 15*Hz* in selected scenarios. The legend is valid for all graphs. RS results for the 100 points random distribution has been omitted as out of a reasonable graph scale at *e* = 0.554 ± 0.004, *u* = 0.0245 ± 0.004 and *v* = 0.44 ± 0.22. Similarly, the CS-WGS uniformity for the same pattern at 2^−8^ compression was omitted, scoring *u* = 0.46 ± 0.17. RS computation times were approximately 25*ms* for 100 spots patterns, and 12*ms* for 36 spots patterns. Error bars report standard deviation.

The computed efficiencies and intensities achievable with a 15 Hz frame rate are reported in Figure 3. Error bars were calculated from the standard deviation of the mean performance over 10 calculations with different initial values of $\theta_{n}^{0}$ and different spatial orientations of the patterns. It can be observed how, for a large amount of regularly spaced spots, WGS has practically no advantage over RS, due to the limited amount of iterations which can be performed within the time limit.

The performance of WGS improve for smaller amounts of spots and less regular patterns, but CS-WGS still stands out as the better performing algorithm in all scenarios. Low compression rates of CS-WGS tend to prioritize uniformity, due to their better sampling of the pupil, while high compression rates tend to prioritize efficiency due to the higher number of iterations achievable. Nonetheless, unless extreme compression factors were used for spots patterns with varying intensities, CS-WGS provides better performance than WGS in all tested scenarios. Results equal or similar to a fully converging implementation of WGS could be achieved in all tested scenarios for well-tuned compression factors.

Since experimental systems are non-ideal, often the performance of the computed patterns can be affected by the experimental setup (Palima and Daria, 2006). In order to prove the improvement in performance provided by CS-WGS is detectable and significant in experimental scenarios, we provided verification of the results of Figure 3 on the setup described in the methods section.

**Figure 4:**
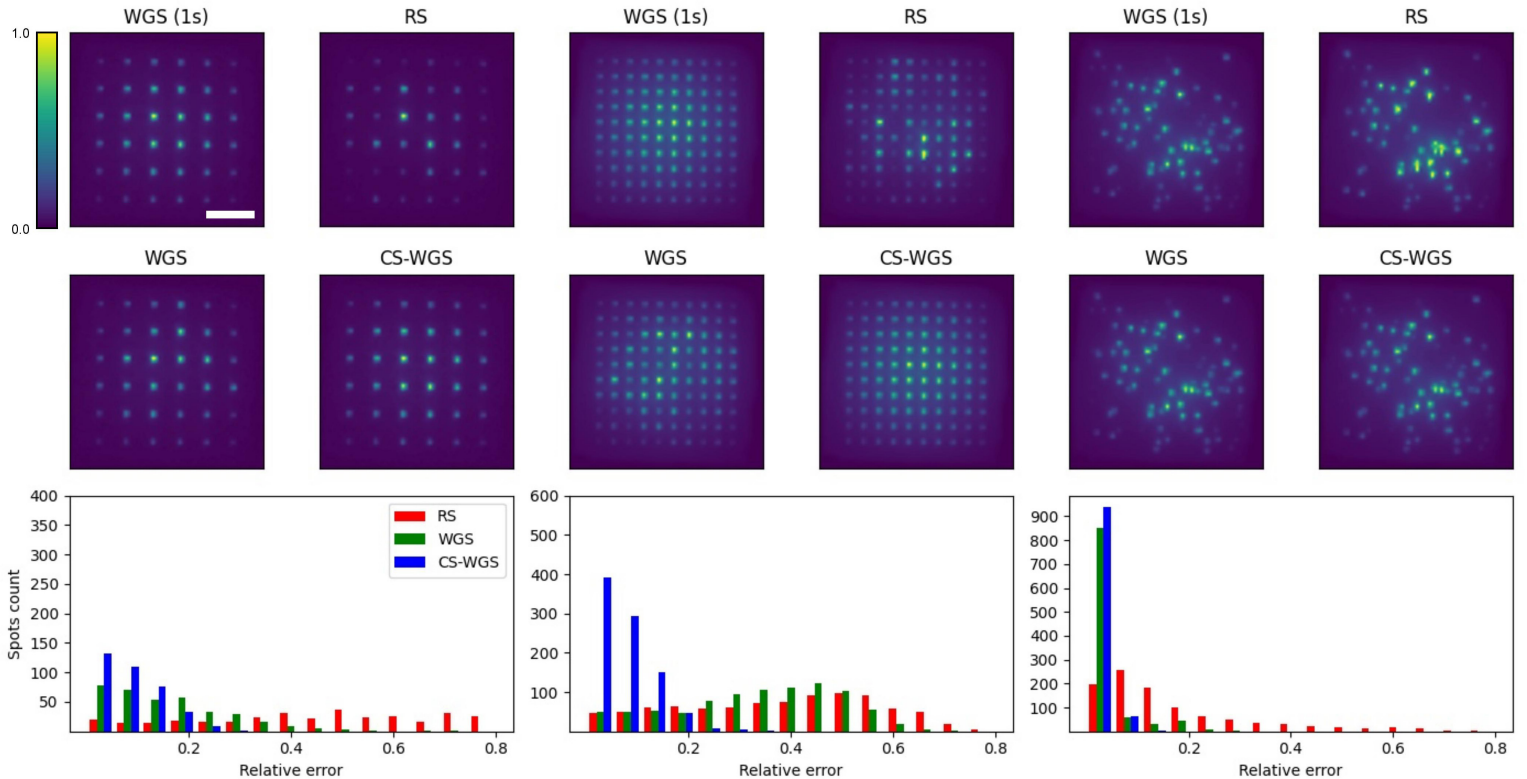
Experimental results, each columns shows representative images for each scenario. Scale bar is 100μ*m*. Scale bar and intensity colorbar are equivalent for all images. It should be noticed how, qualitatively, CS-WGS patterns are practically indistinguishable from full convergence WGS patterns. Significant artifacts can be observed in the grid patterns for both RS and WGS. Significant artifacts are visible for RS in the random spots distribution, while WGS qualitatively seems to perform well. On the bottom row, histograms of the detected spots intensity accuracy over 10 images are shown. It should be observed how CS-WGS does actually outperform significantly WGS, with no spots over 0.25 relative error, while WGS presents outliers up to 0.4.

Experimental results are reported in Figure 4. All holograms show a decrease in signal intensity toward the edges of the frame, due to the loss in diffraction efficiency of the SLM at the edges of its addressable volume, which is independent from the algorithm's performance. Images are reported with a 10X upscaling with bilinear filtering in order to reduce aliased sampling artifacts due to the sensor's low resolution. For each experimental scenario, 10 different variants of the pattern were computed by rotating the grids in three dimensions and rearranging the spots random distribution. In order to estimate the intensity of the spots, a blob detection algorithm was run over images acquired from the camera, integrating the pixels intensities within the blob. blob locations and sizes were estimated over the average of 10 images of the WGS pattern with full convergence and used to compute intensities for the other algorithms. The relative error of each spot's intensity was computed from the ratio of its blob intensity compared to that of the fully converging WGS pattern. It should be noticed the intensity detection error on a fixed pattern could get up to 5% root mean square, depending on the intensity of the spot. As expected, RS performed the worst, with average errors of 0.47 ± 0.30 for the 36 spots grid, 0.32 ± 0.11 for the 100 spots grids, and 0.17 ± 0.02 for the random patterns. When constrained to 64 ms of computation time, WGS performed similarly to RS when computing the 100 spots grid, with an average error of 0.28 ± 0.03, due to its inability to perform more than two iterations in the given time. It performed better for the 100 spots random pattern and the 36 spots grid (respectively 0.03 ± 0.02 and 0.16 ± 0.03 relative errors). Still, CS-WGS proved to provide the best performance in all scenarios, with average errors of 0.08 ± 0.01 for the 36 spots grid, 0.06 ± 0.03 for the 100 spots grids, and 0.02 ± 0.01 for the random patterns. More importantly the highest outliers for all patterns for RS reached relative errors of 0.8, meaning the spot was either almost completely missing or nearly twice as bright as it should have been. Outliers for WGS reached up to 0.8 for the 100 points grid, up to 0.6 for the 36 spots grid and up to 0.4 for the random distribution. Conversely, in all scenarios CS-WGS managed to keep all spots under 0.25 relative error. Computing the same pattern multiple times with different initialization phases led to similar statistics in error distributions. Most importantly, the outlier spots would be positioned in random, unpredictable positions within the pattern.

It should be noted how for the worst case scenario of regular grid patterns, significant deviations from the desired patterns can easily be noticed in the intensity distributions of RS and WGS, while CS-WGS seems indistinguishable from the desired pattern, as highlighted by the numerical metrics. In the random distribution pattern, RS is still visibly inaccurate, while WGS and CS-WGS seem to perform equivalently. However, the numerical metrics highlight how CS-WGS holograms present smaller deviations from the desired pattern, and therefore provide the best achievable performance within the time constraint.

Examples of real time manipulation of the patterns are available as Supplementary Materials, showing the selected patterns rotating in three dimensions through real-time recalculation. The videos show how smooth live update of the hologram is possible, with reasonably constant performance throughout the experiment.

From the results, it is apparent that the compression factor and number of iterations can be fine-tuned to achieve maximum performance. However, this is often not possible for real time generation of generic patterns with varying numbers of spots or geometrical distribution. In such a situation, a compression factor between 1/8 and 1/16 seems to provide a good baseline value to achieve reliable performance in a variety of experimental conditions.

## 4. Discussion

In this manuscript a GPU implementation of the CS-WGS algorithm is presented, and benchmarked against the two most popular alternatives available, being RS and WGS. The results clearly show how the higher convergence speed of CS-WGS, makes it the ideal candidate for real-time applications. The GPU implementation of the algorithm proves, for real time applications, absolutely necessary, as similar spots patterns to those tested would require several seconds for computation with CS-WGS (Pozzi et al., 2019), and up to several minutes with WGS.

While the presented experimental tests were limited by the refresh rate of the available SLM, the algorithm could easily be used to control even faster systems, provided a reasonable amount of spots is selected, and the compression factor is tuned accordingly. The ability of computing high quality holograms in real time could enable real-time, feedback-based control of neuronal networks, driven by calcium (Lu et al., 2017) or voltage activity (Gandolfi et al., 2015) without being limited to stimulation on pre-calculated spatial patterns.

As an example of the advantages of real-time computation compared to the use of pre-computed patterns in closed loop stimulation, keeping *N* cells clamped at the same level of activity through pre-computed patterns by binary switching of photostimulation on each cell, would require pre-calculation of patterns stimulating all possible combinations of at least one of the *N* cells. In practice, this means that 2^*N*^ − 1 patterns would be required, limiting the applicability of the experiment to only a very few neurons.

A similar consideration can be made for the possibility of synchronizing the activity of cell populations to a single “trigger neuron.” For *N* selected trigger neurons, at least 2^*N*^ patterns would need to be calculated, or more if any neuron would need to be coupled with two separate trigger neurons.

It should be acknowledged that fast photoswitching of single points in a given fixed pattern can be achieved by the use of a digital micromirror device in the image plane (Go et al., 2013) to modulate intensity. However, this is still limited in the number of available patterns in the DMD memory (a few tens to a few hundreds, depending on the hardware used), has limited axial positioning extent (only ± 10μm in the reported publication), it would not work for spots located at similar lateral positions but at different axial depths, and in general requires significant modifications to a standard SLM based setup, when compared to a simpler modification of software. Moreover, due to the accuracy of our algorithm in the modulation of power of single spots, even stimulation based on analog modulation of the excitation power for each spot, instead of a binary on/off behavior, could be implemented.

Independently from closed loop photostimulation, an immediate outcome of this implementation lies in the extreme streamlining of the experimental procedure, practically eliminating any waiting time between the selection of the point of interests and the experimental procedure. Of note, it can be extremely useful for *in-vivo* recordings with awake mice. In these circumstances, experiments are in fact extremely time-sensitive, and the minimization of the experiments duration is of utmost importance.

Furthermore, the newly introduced ability of updating the pattern in real-time at the SLM refresh speed limit can potentially enable previously impossible experimental protocols. For instance, the correction of motion artifacts, which is currently performed only through the use of scanners and focus actuators, for rigid linear movements (Vladymyrov et al., 2016), could be enabled for sample rotations and non rigid deformations through SLM patterns adaptation.

Since GPU programming is not a widespread practice amongst the optics and neuroscience research community, the software used to generate the results presented in the paper is made available as a free and open-source library (Pozzi, 2020) for non commercial purposes, to ensure a widespread adoption of the method. The software library is compatible with all SLMs controlled as external screen, and is not necessarily limited to 64 ms computation time. Some modifications to the code may be required to directly drive SLMs with dedicated pci-e interfaces. The software consists in Python (Van Rossum and Drake, 1995) code controlling the GPU using CUDA (Nickolls et al., 2008) through the PyCuda (Klöckner et al., 2012) library and rendering holograms directly to the SLM through the GLFW OpenGL framework.

## Data Availability Statement

The datasets presented in this study can be found in online repositories. The names of the repository/repositories and accession number(s) can be found in the article/Supplementary Material.

## Author Contributions

PP designed the research, performed the experiments, and wrote the first version of the manuscript. JM designed the research and contributed to the manuscript writing. All authors contributed to the article and approved the submitted version.

## Conflict of Interest

The authors declare that the research was conducted in the absence of any commercial or financial relationships that could be construed as a potential conflict of interest.
