# Supplementary figures and images for: Real Time Generation of Three Dimensional Patterns for Multiphoton Stimulation
Source: Front Cell Neurosci. 2021 Feb 24;15:609505. doi: 10.3389/fncel.2021.609505 (PMC7943733; doi:10.3389/fncel.2021.609505)

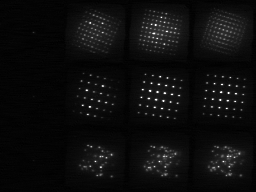

Supplement: Supplementary file 2 [file Image_1.TIF]
